# Higher Arm Versus Lower Arm Systolic Blood Pressure and Cardiovascular Outcomes: a Meta-Analysis of Individual Participant Data From the INTERPRESS-IPD Collaboration

**DOI:** 10.1161/HYPERTENSIONAHA.121.18921

**Published:** 2022-08-02

**Authors:** Christopher E. Clark, Fiona C. Warren, Kate Boddy, Sinéad T.J. McDonagh, Sarah F. Moore, Maria Teresa Alzamora, Rafel Ramos Blanes, Shao-Yuan Chuang, Michael H. Criqui, Marie Dahl, Gunnar Engström, Raimund Erbel, Mark Espeland, Luigi Ferrucci, Maëlenn Guerchet, Andrew Hattersley, Carlos Lahoz, Robyn L. McClelland, Mary M. McDermott, Jackie Price, Henri E. Stoffers, Ji-Guang Wang, Jan Westerink, James White, Lyne Cloutier, Rod S. Taylor, Angela C. Shore, Richard J McManus, Victor Aboyans, John L. Campbell

**Affiliations:** Primary Care Research Group, University of Exeter Medical School, Exeter, Devon, England (C.E.C., F.C.W., S.T.J.M., S.F.M., R.S.T., J.L.C.).; Patient and Public Involvement Team, PenCLAHRC, University of Exeter Medical School, South Cloisters, Exeter, Devon, England (K.B.).; Unitat de Suport a la Recerca Metropolitana Nord, Fundació Institut Universitari per a la recerca a l’Atenció Primària de Salut Jordi Gol i Gurina (IDIAPJGol), Mataró, Spain (M.T.A.).; Unitat de Suport a la Recerca Girona. Fundació Institut Universitari per a la recerca a l’Atenció Primària de Salut Jordi Gol i Gurina (IDIAPJGol), Institut d’Investigació Biomèdica de Girona (IdIBGi), Department of Medical Sciences, School of Medicine, University of Girona, Spain (R.R.B.).; Institute of Population Health Sciences, National Health Research Institutes (NHRI), Zhunan, Taiwan, ROC (S.-Y.C.).; Department of Family Medicine and Public Health, University of California, San Diego, School of Medicine, La Jolla, CA (M.H.C.).; Vascular Research Unit, Department of Vascular Surgery, Viborg Regional Hospital, Denmark and Department of Clinical Medicine, Aarhus University (M.D.).; Department of Clinical Science in Malmö, Lund University, Sweden (G.E.).; Institute of Medical Informatics, Biometry and Epidemiology, University Hospital Essen, Essen, Germany (R.E.).; Division of Gerontology and Geriatric Medicine, Wake Forest School of Medicine, NC (M.E.).; National Institute on Aging, Baltimore, MD (L.F.).; INSERM, Univ. Limoges, CHU Limoges, IRD, U1094 Tropical Neuroepidemiology, Institute of Epidemiology and Tropical Neurology, GEIST, Faculté de Médecine de l’Université de Limoges - 2 rue du Dr Marcland - 87 025 Limoges Cedex, France (M.G., V.A.).; Institute of Biomedical and Clinical Science, University of Exeter Medical School, RILD, Exeter, Devon, England (A.H.).; Lípid and Vascular Risk Unit. Internal Medicine Service, Carlos III - La Paz Hospital, Madrid, Spain (C.L.).; Department of Biostatistics, University of Washington (R.L.M.).; Northwestern University Feinberg School of Medicine, Chicago, IL (M.M.M.).; Usher Institute of Population Health Sciences and Informatics, University of Edinburgh, Scotland (J.P.).; Department of Family Medicine, CAPHRI Care and Public Health Research Institute, Maastricht University, The Netherlands (H.E.S.).; Centre for Epidemiological Studies and Clinical Trials, Shanghai Key Laboratory of Hypertension, The Shanghai Institute of Hypertension, Ruijin Hospital, Shanghai Jiaotong University School of Medicine, China (J.-G.W.).; Department of Vascular Medicine, University Medical Center Utrecht, The Netherlands (J.W.).; DECIPHer, Centre for Trials Research, College of Biomedical and Life Sciences, Cardiff University, Cardiff (J.W.).; Département des sciences infirmières, Université du Québec à Trois-Rivières, Trois-Rivières, Québec, Canada (L.C.).; MRC/CSO Social and Public Health Sciences Unit & Robertson Centre for Biostatistics, Institute of Health and Well Being, University of Glasgow (R.S.T.).; NIHR Exeter Clinical Research Facility, Royal Devon and Exeter Hospital and University of Exeter, England (A.C.S.).; Nuffield Department of Primary Care Health Sciences, University of Oxford, England (R.J.M.).; Department of Cardiology, Dupuytren University Hospital, and Inserm 1094, Tropical Neuroepidemiology, Limoges, France (V.A.).

**Keywords:** blood pressure, cardiovascular diseases, hypertension, meta-analysis

## Abstract

Guidelines recommend measuring blood pressure (BP) in both arms, adopting the higher arm readings for diagnosis and management. Data to support this recommendation are lacking. We evaluated associations of higher and lower arm systolic BPs with diagnostic and treatment thresholds, and prognosis in hypertension, using data from the Inter-arm Blood Pressure Difference—Individual Participant Data Collaboration.

**METHODS:**

One-stage multivariable Cox regression models, stratified by study, were used to examine associations of higher or lower reading arm BPs with cardiovascular mortality, all-cause mortality, and cardiovascular events, in individual participant data meta-analyses pooled from 23 cohorts. Cardiovascular events were modelled for Framingham and atherosclerotic cardiovascular disease risk scores. Model fit was compared throughout using Akaike information criteria. Proportions reclassified across guideline recommended intervention thresholds were also compared.

**RESULTS:**

We analyzed 53 172 participants: mean age 60 years; 48% female. Higher arm BP, compared with lower arm, reclassified 12% of participants at either 130 or 140 mm Hg systolic BP thresholds (both *P<*0.001). Higher arm BP models fitted better for all-cause mortality, cardiovascular mortality, and cardiovascular events (all *P*<0.001). Higher arm BP models better predicted cardiovascular events with Framingham and atherosclerotic cardiovascular disease risk scores (both *P*<0.001) and reclassified 4.6% and 3.5% of participants respectively to higher risk categories compared with lower arm BPs).

**CONCLUSIONS:**

Using BP from higher instead of lower reading arms reclassified 12% of people over thresholds used to diagnose hypertension. All prediction models performed better when using the higher arm BP. Both arms should be measured for accurate diagnosis and management of hypertension.

**REGISTRATION:**

URL: https://www.clinicaltrials.gov; Unique identifier: CRD42015031227.

**Novelty and Relevance**

**What Is New?**

- Guidelines advise adoption of the higher reading arm blood pressure for diagnosis and management of hypertension, based on expert opinion. We studied data on over 53 000 participants from 23 studies around the world to examine the implications of choosing the higher or lower arm blood pressure.
- The higher arm blood pressure better predicts all-cause mortality, cardiovascular mortality, and cardiovascular events, compared with the lower arm reading.
- Up to 12% of people classified with hypertension fall below recommended thresholds for diagnosis and treatment of hypertension if the lower, rather than the higher reading arm, is used.

**What Is Relevant?**

- Routine adoption of the higher arm blood pressure measurement allows better cardiovascular risk prediction and classification, more accurate diagnosis and treatment of hypertension

**Clinical/Pathophysiological Implications?**

- When considering diagnosis or treatment of hypertension, blood pressure should be measured in both arms and all decisions based on the higher arm measurement. Failure to do this risks under-diagnosis and under-treatment for many millions of people globally.

Differences in blood pressure readings between arms have been recognised for over a century. This raises the question of which arm blood pressure—the higher or the lower arm reading—should be routinely adopted for the diagnosis and management of hypertension? International guidelines advise checking blood pressure in both arms, when assessing people for hypertension, and adoption of the higher arm reading where differences exist; this is, however, based on expert consensus opinion alone.^1–3^ Only 20% to 50% of patients in primary care reportedly have both arms measured and general practitioners do not always adopt the higher reading arm for management decisions.^4,5^ Inter-arm differences may arise due to stenosis, making adoption of the higher arm reading logical; however, in most instances, there is no clearly demonstrable stenosis.^6^ Studies that compare the prognostic values associated with higher and lower reading arm blood pressures are lacking.

This study examined data from the Inter-arm Blood Pressure Difference—Individual Participant Data (INTERPRESS-IPD) Collaboration.^7^ Individual-level data on systolic blood pressure in each arm allowed us to compare associations of the higher and lower arm systolic blood pressures with cardiovascular events, mortality, and hypertension diagnoses. Since treatment decisions for primary prevention of cardiovascular events are based on cardiovascular risk score thresholds, the impact of using the higher compared to the lower arm reading on reclassification across intervention thresholds was also examined.

## Methods

Data supporting the findings of this study are available from the corresponding author upon reasonable request. The INTERPRESS-IPD Collaboration was established to examine the independent associations of systolic inter-arm difference (IAD) with cardiovascular events, all-cause mortality, and cardiovascular mortality using individual participant data (IPD) meta-analyses and has been described in detail previously.^7,8^ In brief, systematic searches for prospective studies that recorded systolic blood pressure in both arms at recruitment were undertaken in Medline, Old Medline, Medline in process, Embase, and CINAHL databases until January 2017; unpublished data were also sought. Inclusion criteria were participants aged ≥18 years, recruited from community, primary care, or general clinic settings. Required primary outcomes were one or more of all-cause mortality, cardiovascular mortality, or fatal and nonfatal cardiovascular events.

Studies were independently selected by 2 authors using *Covidence* (Veritas Health Innovation, Melbourne, Australia). Disagreements were resolved by discussion between authors. Lead authors of potentially eligible studies were invited to participate, with a data sharing agreement and pro-forma request for data required. Nonresponders received 2 further invitations.

Anonymised datasets were cleaned individually and then combined to form a single dataset for the INTERPRESS-IPD Collaboration. Analyses were undertaken using (Stata v17.0, Statacorp, TX).

For these analyses, we used the first pair of left and right arm systolic blood pressure readings. Reclassification across clinically important blood pressure thresholds, according to choice of arm, was examined. Follow-up was truncated at 10 years to minimize censorship while permitting comparison of 10-year outcomes for risk scores. Baseline descriptive data were calculated for all participants with available data; no imputation of missing data was performed. Multilevel mixed-effects linear regression models, with a random effect for study, were used to compare values based on higher and lower arms. Due to low between-study heterogeneity within the dataset, which caused failure of previous 1-stage random effects flexible parametric models to converge, we used fixed effect 1-stage Cox proportional hazards modelling, stratified by study.^7,8^ We compared models based on higher or lower arm blood pressures alone, then adjusted for age and sex. Finally, we compared a fully adjusted model previously derived and validated in the INTERPRESS-IPD dataset.^7^ This model adjusted for age, sex, current smoking status, ethnicity, total cholesterol and preexisting diagnoses of diabetes and/or hypertension. In sensitivity analyses, we added terms for preexisting cardiovascular disease and/or renal disease to the fully adjusted model to explore differences associated with higher preexisting risks of mortality and cardiovascular events.

For participants free of preexisting cardiovascular disease, we compared the atherosclerotic cardiovascular disease (ASCVD) scores and Framingham risk scores for 10-year risks of cardiovascular events.^9,10^ The European Systematic Coronary Risk Evaluation (SCORE) score for cardiovascular mortality was also examined. However, it has recently been superceded by SCORE2; therefore, these results are summarised, but presented in full in the Supplemental Material.^11,12^ We calculated each score from the higher and lower arm blood pressures. We restricted analyses to participants within age ranges and ethnicities applicable to each risk score (ASCVD: 40–79, Framingham: 20–79 and SCORE: 40–65 years).^9–11^ We assessed classification across the American (10%), Canadian (15%), and European (1%, 5%, and 10%) guideline recommended 10-year cardiovascular risk thresholds.^1–3^

More than 1 pair of blood pressure readings were available for a minority of participants. We conducted a sensitivity analysis of the primary outcomes, and risk score categorization, using the mean of all available pairs of recruitment blood pressures to account for repeated measures. To specifically address implications for clinically important inter-arm systolic blood pressure differences,^7^ we also repeated the primary outcomes and risk score categorisation restricted to participants with a systolic IAD ≥ 10 mm Hg.

Regression model fit using higher or the lower reading arm blood pressures was compared using the Akaike information criterion (AIC).^13^ This measures the information lost when approximating reality, thus qualitatively model fit is considered better with a lower AIC estimate indicating improved risk prediction. As a rule of thumb a difference in AIC (ΔAIC) between models <2 indicates that they are comparable, ΔAIC between 4 and 7 indicates considerably less support, and ΔAIC >10 essentially no support for the likelihood that the higher scoring model is better.^14^ To facilitate interpretation, raw differences in AIC between arm models were assigned probabilities using Akaike weights.^15^ Likelihood ratios were also compared, and concordance of models assessed with Harrell *C* statistic.^16^

Data-sharing agreements were signed with each collaborating study lead author. All data were originally collected under cohort specific ethical approvals so further approval was not required for this IPD meta-analysis. This study was conducted in accordance with the Preferred Reporting Items for Systematic Reviews and Meta-Analyses of IPD (PRISMA-IPD) statement.^17^ The protocol is registered at PROSPERO (record no: CRD42015031227).

## Results

The current analyses were conducted using 53 172 participants with blood pressure recorded in both arms from 23 cohorts in the INTERPRESS-IPD Collaboration (Table 1; Table S1). Mean age was 60 (SD 12.5) years and 48% were female. The mean difference in systolic blood pressure between higher and lower reading arms was 6.6 mm Hg ([95% CI, 0.0–20.0]; *P*<0.001; Table 1; Figure). Using the higher arm measurement compared with using the lower arm resulted in reclassification of 6572 (12.4%) of participants’ systolic blood pressures from below to above 130 mm Hg, and 6339 (11.9%) from below to above 140 mm Hg (*P*<0.001 for both).

**Table 1.**
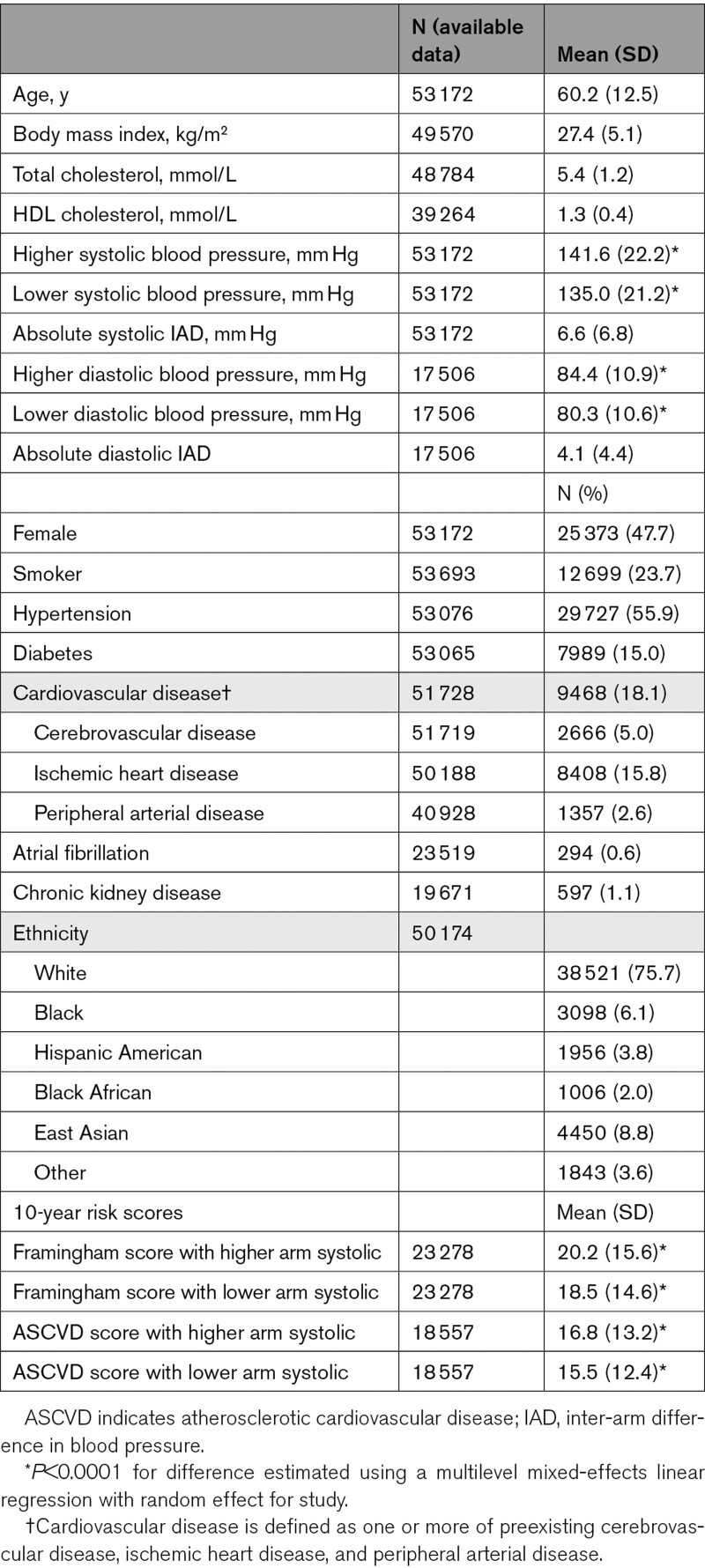
Baseline Characteristics of the Cohort

**Figure.**
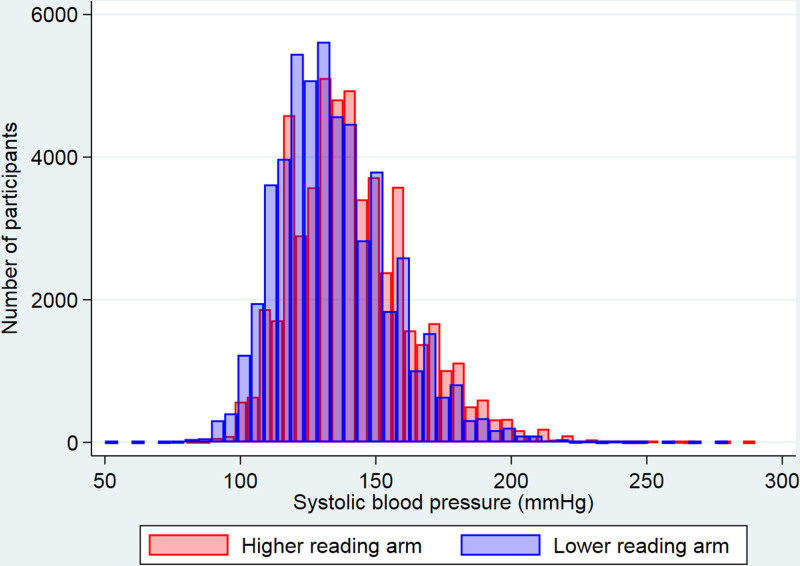
Distribution of higher and lower reading arm systolic blood pressures.

All-cause and cardiovascular mortality outcome data were available for 50 009 participants pooled from 22 cohorts with 4810 (9.6%) deaths; 1 cohort only collected a combined fatal and nonfatal outcome. For all-cause mortality, all models returned lower AIC values when based on the higher arm systolic blood pressure compared with the lower reading arm. The weighted difference in AICs was significant in all analyses, except for the sensitivity analysis with preexisting renal disease. Harrell’s *C* statistics and likelihood ratio tests were also consistently in favor of models based on the higher compared with the lower arm (Table 2). Findings were similar for cardiovascular mortality, with significant differences for all models in favor of the higher reading arm blood pressure.

**Table 2.**
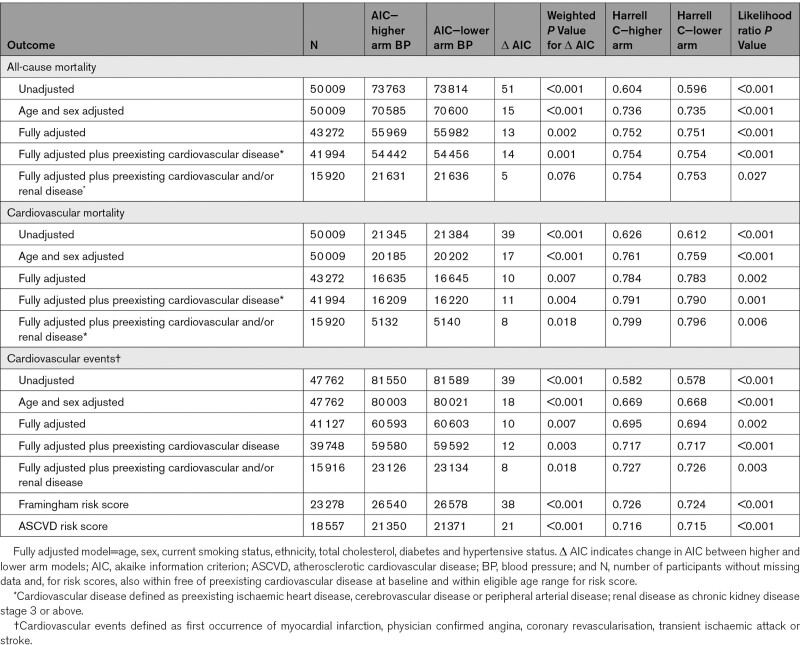
Model Comparisons for All-Cause Mortality, Cardiovascular Mortality, and Cardiovascular Events Based on Higher and Lower Reading Arms

Mean European SCORE values for 18 017 eligible participants free of preexisting cardiovascular disease were significantly higher when calculated using the higher arm blood pressure in comparison to the lower arm reading (2.6 [SD 2.8] versus 2.3 [2.4]; *P*<0.001). However, model fit according to higher or lower arm did not differ (Supplemental Material).

Fatal and nonfatal cardiovascular events were recorded for 47 762 participants pooled from 22 cohorts; one other cohort did not record nonfatal events. All models returned substantially lower AIC values when the higher arm systolic blood pressure, compared with the lower arm, was used in the model. Harrell *C* statistics suggested improved model concordance at each adjustment and were consistently better for higher arm models compared with equivalent lower arm models. Likelihood ratio tests were also significant for all comparisons.

Use of higher compared with the lower arm systolic blood pressure resulted in participants being reclassified into clinically important treatment groups. Mean Framingham risk scores for 23 278, and ASCVD for 18 557 eligible participants, were significantly higher when calculated using the higher arm systolic blood pressure in comparison to the lower arm reading (*P*<0.001; Table 1). AIC values were significantly lower, Harrell *C* statistics higher, and likelihood ratios significant for both scores in favor of models calculated from the higher arm systolic blood pressure (Table 2). Reclassification from below to above guideline-recommended risk thresholds, when higher rather than lower arm blood pressures were used, occurred for 4.6% of participants with Framingham scores and 3.5% with ASCVD scores (*P*<0.001 for both; Table 3).

**Table 3.**
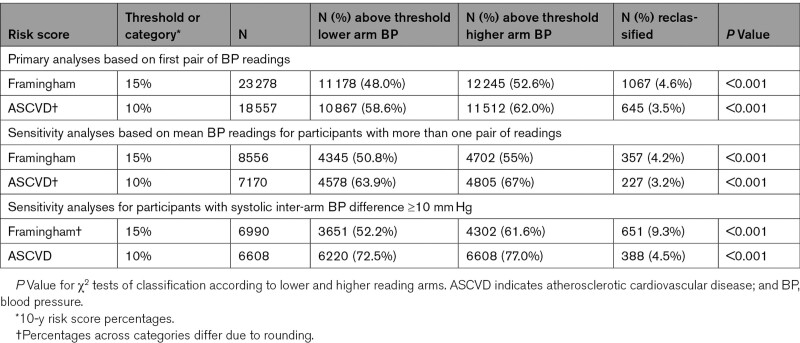
Reclassification Across Guideline-Recommended Risk Thresholds for Commonly Used Cardiovascular Risk Scores

More than one pair of systolic blood pressures was recorded for 12 861 participants in the INTERPRESS-IPD dataset. Differences between higher and lower arm models were smaller using mean values, but AIC remained lower in all unadjusted models, with likelihood ratio *P* <0.05 for all 3 primary outcomes. Differences were no longer significant in any adjusted models; however, rates of reclassification across risk thresholds remained similar to the single pair estimates (Table 3). For small inter-arm differences, the side of the higher arm reading could fluctuate over 2 pairs of measurements between right and left. Concordance of the higher side was 63% overall, rising to 73% when mean IAD was ≥5 mmHg and 92% for ≥10 mmHg.

There were 15 038 (28.3%) participants with a systolic IAD≥10 mm Hg, based on a single pair of readings. Higher compared with lower arm blood pressure measurement resulted in reclassification of 3644 (24.2%) of these participants’ systolic blood pressures from below to above 130 mm Hg, and 3784 (25.2%) from below to above 140 mm Hg (*P*<0.001 for both). Reclassification, from below to above guideline-recommended risk thresholds within this subgroup, was observed for 9.3% of participants with Framingham scores and 4.5% with ASCVD scores (*P*<0.001 for both; Table 3).

## Discussion

This IPD meta-analysis found that using the higher reading arm blood pressure, in comparison to the lower reading arm, reclassified 12% of people against thresholds for diagnosing hypertension currently recommended in American, Canadian, and European guidelines. Adoption of the higher reading arm resulted in better fits for models of all-cause mortality, cardiovascular mortality, and cardiovascular morbidity, when compared with the lower reading arm. Use of blood pressure readings from the higher compared with the lower reading arm consistently improved fit for models using the established Framingham and ASCVD risk scores. Approximately 4% of people were reclassified from below to above clinically important Framingham or ASCVD cardiovascular risk thresholds when the higher rather than the lower arm blood pressure was used. Such reclassification would have resulted in participants being offered guideline recommended blood pressure and/or cholesterol-lowering medications.

Current and previous hypertension guidelines recommend that both arms are measured during blood pressure assessment.^1–3^ However, no published evidence in support of these consensus views has been identified. Surveys suggest that implementation of these recommendations is less than satisfactory.^4^ In the United Kingdom, 25% of general practitioners do not routinely adopt the higher reading arm for decisions concerning blood pressure.^5^ If reproducible, the findings presented here suggest that many millions of people should be reclassified into higher cardiovascular risk and blood pressure control categories that would warrant treatment. Acting on this evidence would have major effects on cardiovascular risk by optimization of treatments being offered and/or targets set.^18^

These findings are immediately applicable to, and of relevance for, anyone who undergoes routine measurement or treatment of high blood pressure. The results quantify the potential clinical importance of a failure to identify the higher reading arm. Such an omission risks errors in blood pressure interpretation, the diagnosis of hypertension, and sub-optimal blood pressure control.^19,20^ For the last 80 years, international hypertension guidelines have recommended the use of the higher reading arm based on expert consensus.^1,2,21–23^ Nevertheless, in practice, this guidance is not always followed.^5^ Proportions of people with a higher reading right or left arm are almost equal, so blood pressure must be measured in both arms to identify the higher reading arm. Arm dominance has a minor impact on distribution, but it cannot be assumed that either the right or the left arm is systematically the higher.^24^ Failure to recognize the higher arm could misclassify up to 12% of our participants to below rather than above clinically important blood pressure thresholds, and between 3% and 8% erroneously below cardiovascular risk thresholds. This presents a clear risk of underestimation and therefore undertreatment of cardiovascular risk through failure to intensify primary prevention treatments appropriately. We have previously shown that adjusted all-cause mortality begins to rise with a systolic IAD≥5 mmHg.^7^ Where analyses were restricted to participants with a systolic IAD≥10 mm Hg, blood pressure was reclassified across treatment thresholds for up to 25%, and up to 9% were reclassified across cardiovascular risk thresholds. These higher figures emphasise the proportionally greater risks of misclassification when a significant IAD is not detected by failure to measure both arms.

### Study Limitations

The INTERPRESS-IPD Collaboration was formed to investigate the associations of interarm blood pressure differences with mortality, uniquely bringing together prospective survival data for over 50 000 participants with blood pressure recorded in both arms. Participants were included from North America, Europe, Asia, and Central Africa, thus representing a range of ethnicities.^7^ To ensure clinically relevant findings, openly available cardiovascular risk prediction algorithms used in practice in Europe, United States, and Canada were chosen for these analyses.

Hypertension is generally diagnosed using multiple rather than single blood pressure readings. The primary analyses in this study were based on single pairs of measurements to maximize use of available data and relevance to routine primary care. Regression to the mean reduced differences between mean lower and higher arm blood pressures; however, classification above guideline recommended thresholds remained statistically and clinically higher when mean higher arm blood pressures were used. Considerable data cleaning and preparation were required to pool data from included cohorts for these analyses, leading inevitably to passage of time since closure of data collection in 2017. While this may have excluded relevant more recent publications from inclusion in the Collaboration, search updates continued to April 2020 without identifying any additional clearly eligible studies. Since the dataset offered ample power to address the research questions posed here, it seems unlikely that the findings would be sensitive to further inclusion of any more recent additional data.

### Conclusions

This study provides robust evidence that more than 1 in 10 people were recategorized to require additional treatment by using the higher rather than the lower reading arm. Blood pressure should be assessed in both arms and readings from the higher reading arm should be used in the diagnosis and management of hypertension and cardiovascular disease. This choice also significantly improved the prognostic ability of blood pressure measurement for cardiovascular events. Failure to use the higher reading arm risks underdiagnosis, undertreatment of high blood pressure and under-estimation of cardiovascular risk for many millions worldwide, missing opportunities to appropriately intensify primary prevention of cardiovascular disease.

### Perspectives

Implications of routinely adopting the higher reading arm blood pressure measurement, as opposed to the lower arm reading, were investigated in a multinational individual participant data meta-analyses of over 50 000 participants. In a range of models, the higher reading arm blood pressure consistently performed better in predicting 10-year outcomes for all-cause mortality, cardiovascular mortality, and cardiovascular events. Similarly, Framingham and ASCVD scores modelled cardiovascular event risks better when calculated using the higher reading arm. When compared with lower arm blood pressure readings, the higher arm reclassified 12% of people from below to above thresholds for diagnosing hypertension currently recommended in American, Canadian, and European guidelines.

Blood pressure should be measured in both arms and the higher reading adopted for hypertension diagnosis and management. Failure to use the higher reading arm risks underdiagnosis, undertreatment of high blood pressure and under-estimation of cardiovascular risk for many millions worldwide, missing opportunities to appropriately intensify primary prevention of cardiovascular disease.

## ARTICLE INFORMATION

### Acknowledgments

We thank our Independent Monitoring Group: Alun Hughes (chair), Gary Collins and Tom Fahey; Data management Group: Tim Eames and Sofia Sanabria; Administrators: Ellie Kingsland and Chloe Thomas.

### Author Contributions

C.E. Clark proposed the current analyses; C.E. Clark, K. Boddy, F.C. Warren, V. Aboyans, L. Cloutier, R.J. McManus, A.C. Shore, R.S. Taylor, and J.L. Campbell were co-applicants on the INTERPRESS-IPD grant; SMcD and SM are researchers on the project. KB led public and patient involvement throughout the study. S.T.J. McDonagh, S.F. Moore, J.L. Campbell, A.C. Shore, and C.E. Clark undertook literature searches, C.E. Clark negotiated data access with collaborating authors. F.C. Warren led data cleaning, initial analyses, and supervised current analyses. C.E. Clark undertook analyses presented here. A.C. Shore, L. Cloutier, R.J. McManus, R.S. Taylor, and V. Aboyans advised on study conduct, analysis, and interpretation of findings. All authors contributed to development of the article and read and reviewed the final article. C.E. Clark has full access to data and acts as guarantor for this study.

### Sources of Funding

The INTERPRESS-IPD Collaboration was funded by the National institute for Health Research (NIHR) Research for Patient Benefit Programme (PB-PG-0215-36009). C.E. Clark is funded by NIHR School for Primary Care Research (SPCR) Grant (Ref: 512). S.T.J. McDonagh is funded by NIHR SPCR Fellowship. R.J. McManus is supported by NIHR Oxford CLAHRC. A.C. Shore is supported by NIHR Exeter Clinical Research Facility. J.-G. Wang supported by grants from National Natural Science Foundation of China (91639203 and 82070435), Beijing and Shanghai Commission of Science and Technology (19DZ2340200), Shanghai, China. Views expressed are those of the authors and not necessarily those of the NIHR, the NHS, the Department of Health and Social Care or the funding bodies acknowledged.

### Disclosures

C.E. Clark received honoraria from Bayer and ReCor medical for unrelated work. R.L. McClelland received monitors for research from Omron and works with them on a telemonitoring project. J.-G. Wang received lecture and consulting fees from Novartis, Omron, Servier and Takeda. No company had, or will have, any involvement in design, conduct or reporting of this study.

## Supplementary Material


