# Supplementary material for: Higher Arm Versus Lower Arm Systolic Blood Pressure and Cardiovascular Outcomes: a Meta-Analysis of Individual Participant Data From the INTERPRESS-IPD Collaboration
Source: Hypertension. 2022 Aug 2;79(10):2328–35. doi: 10.1161/HYPERTENSIONAHA.121.18921 (PMC9444257; doi:10.1161/HYPERTENSIONAHA.121.18921)
Supplement: Supplementary file 1 [file hyp-79-2328-s001.pdf]

Higher arm versus lower arm systolic blood pressure and cardiovascular outcomes: A meta-analysis of individual participant data from the INTERPRESS-IPD Collaboration

Data Supplement

Christopher E Clark PhD MBChB FRCP[1], Fiona C Warren PhD[1], Kate Boddy MSc[2], Sinéad TJ McDonagh PhD[1], Sarah F Moore MB BChir[1], Maria Teresa Alzamora PhD[3], Rafel Ramos Blanes MD PhD[4], Shao-Yuan Chuang PhD[5], Michael H Criqui MD, MPH[6], Marie Dahl RN PhD[7], Gunnar Engström MD PhD[8], Raimund Erbel MD[9], Mark Espeland PhD[10], Luigi Ferrucci MD, PhD [11], Maëlenn Guerchet PhD[12], Andrew Hattersley DM[13], Carlos Lahoz MD PhD[14], Robyn L McClelland PhD[15], Mary M McDermott MD[16], Jackie Price MD[17], Henri E Stoffers MD PhD [18], Ji-Guang Wang MD PhD[19], Jan Westerink MD PhD[20], James White PhD[21], Lyne Cloutier RN PhD[22], Rod S Taylor PhD[1 &23], Angela C Shore PhD[24], Richard J McManus PhD MBBS FRCGP[25], Victor Aboyans MD PhD FAHA [12,& 26], John L Campbell MD MBChB FRCGP[1]

1. Primary Care Research Group, Institute of Health Services Research, University of Exeter Medical School, College of Medicine & Health, Smeall Building, St Luke's Campus, Magdalen Road, Exeter, Devon, England, EX1 2LU

2. Patient and Public Involvement Team, PenCLAHRC, University of Exeter Medical School, College of Medicine & Health, South Cloisters, St Luke's Campus, Magdalen Road, Exeter, Devon, England, EX1 2LU

3. Unitat de Suport a la Recerca Metropolitana Nord, Fundació Institut Universitari per a la recerca a l'Atenció Primària de Salut Jordi Gol i Gurina (IDIAPJGol), Mataró, Spain

4. Unitat de Suport a la Recerca Girona. Fundació Institut Universitari per a la recerca a l'Atenció Primària de Salut Jordi Gol i Gurina (IDIAPJGol), Institut d'Investigació Biomèdica de Girona (IdIBGi), Department of Medical Sciences, School of Medicine, University of Girona, Girona, Spain

5. Institute of Population Health Sciences, National Health Research Institutes (NHRI), No35. Keyan Road, Zhunan, Miaoli County 35053, Taiwan, R.O.C

6. Department of Family Medicine and Public Health, University of California, San Diego, School of Medicine, 9500 Gilman Drive, La Jolla, CA, 92093-0628, USA

7. Vascular Research Unit, Department of Vascular Surgery, Viborg Regional Hospital, Heibergs Allé 4, 8800 Viborg, Denmark and Department of Clinical Medicine, Aarhus University, Palle Juul-Jensens Blvd. 82, 8200 Aarhus N, Denmark

8. Department of Clinical Science in Malmö, Lund University, CRC 60:13, Box 50332, 20213 Malmö, Sweden

9. Institute of Medical Informatics, Biometry and Epidemiology, University Hospital Essen, Hufelandstraße 55, D-45147 Essen, Germany

10. Division of Gerontology and Geriatric Medicine, Wake Forest School of Medicine, North Carolina, USA

11. National Institute on Aging, 251 Bayview Boulevard, Baltimore MD 21224, USA

12. INSERM U1094 & IRD, Tropical Neuroepidemiology, Institut d'Epidémiologie et de Neurologie Tropicale (IENT), Faculté de Médecine de l'Université de Limoges - 2 rue du Dr Marcland - 87 025 Limoges Cedex, France

13. Institute of Biomedical and Clinical Science, University of Exeter Medical School, College of Medicine and Health, RILD, Barrack Road, Exeter, Devon, England, EX2 5DW

14. Lípid and Vascular Risk Unit. Internal Medicine Service. Carlos III - La Paz Hospital. Madrid. Spain.

15. Department of Biostatistics, University of Washington, Washington, USA

16. Northwestern University Feinberg School of Medicine, 750 North Lake Shore Drive, 10th floor, Chicago, IL b60611, USA

17. Usher Institute of Population Health Sciences and Informatics, University of Edinburgh, Scotland, EH8 9AG

18 Department of Family Medicine, CAPHRI Care and Public Health Research Institute, Maastricht University, P.O.Box 616, 6200 MD Maastricht, The Netherlands

19. Centre for Epidemiological Studies and Clinical Trials, Shanghai Key Laboratory of Hypertension, The Shanghai Institute of Hypertension, Ruijin Hospital, Shanghai Jiaotong University School of Medicine, Ruijin 2nd Road 197, Shanghai 200025, China

20. Department of Vascular Medicine, University Medical Center Utrecht, Utrecht, The Netherlands

21. DECIPHer, Centre for Trials Research, College of Biomedical and Life Sciences, Cardiff University, 4th Floor, Heath Park, Cardiff, CF14 4YS

22. Département des sciences infirmières, Université du Québec à Trois-Rivières, 3351 Boulevard des Forges, Trois-Rivières, Québec, Canada, G9A5H7

23. MRC/CSO Social and Public Health Sciences Unit & Robertson Centre for Biostatistics, Institute of Health and Well Being, University of Glasgow

24. NIHR Exeter Clinical Research Facility, Royal Devon and Exeter Hospital and University of Exeter College of Medicine & Health, Barrack Road, Exeter, Devon, England, EX2 5AX

25. Nuffield Department of Primary Care Health Sciences, University of Oxford, Radcliffe Primary Care Building, Radcliffe Observatory Quarter, Woodstock Road, Oxford, England, OX2 6GG

26. Department of Cardiology, Dupuytren University Hospital, and Inserm 1094, Tropical Neuroepidemiology, Limoges, France

Content

Description of the INTERPRESS-IPD Collaboration cohort

Table S1 – Characteristics of included studies

References to included studies

Results for SCORE analyses

Table S2 - Reclassification across guideline recommended risk thresholds for SCORE cardiovascular mortality score

Data sources and funding acknowledgements for data contributing to the INTERPRESS-IPD Collaboration

Description of the INTERPRESS-IPD Collaboration cohort

In establishing the INTERPRESS-IPD Collaboration, 4,448 unique titles and abstracts were screened, and 152 full texts assessed for eligibility. Authors of 60 potentially relevant cohorts were contacted leading to exclusion of 13 on clarification of methodological queries; another 13 did not respond to repeated invitations and a further ten author groups were unable to supply data. In all 24 individual participant datasets were obtained totalling 57,434 records. One dataset contained inter-arm differences rather than pairs of bilateral blood pressures, therefore, the current analyses were conducted using 53,172 participants from 23 cohorts in the INTERPRESS-IPD Collaboration, with a record of blood pressure measured in both arms.[1-23]

Participants originated from Western Europe (14 cohorts), USA (6), East Asia (2) and Central Africa (1). The majority (76%) of participants were White, with lower representation of East Asian, African American, Hispanic American and Black African ethnicities (Table 1; Data Supplement: Table S1).

Table S1 – Characteristics of included studies

Study name	Period of patient recruitment /Duration of trial	Sample size	Country of origin	Eligibility criteria	Primary outcome measure	Blood pressure measurement method for IAD	Intended maximum duration of follow up	Definition of hypertension	Definition of diabetes	Definition of cardiovascular death and non-fatal cardiovascular event
Aspirin in Asymptomatic Atherosclerosis (AAA)[1]	April 1998-October 2008	3350	Scotland	Males and females, aged 50-75 years, living in central Scotland, free of clinical cardiovascular disease with an ABI < 0.95	Initial fatal or non-fatal coronary event or stroke or revascularisation	Single pair of sequential BP readings recorded using a Doppler probe (Huntleigh Healthcare, Cardiff) and aneroid desk sphygmomanometer (Accoson;A.C. Cossor Ltd, London, UK) with patient supine	5 years with extended follow-up of 4.5 years. Mean 8.2 years	N/S	Self-reported diabetes	*Cardiovascular death*: Definite or probable fatal MI, death due to IHD, or fatal stroke due to infarction.<br><br>*Non-fatal events*: MI, stroke or TIA, coronary or peripheral revascularisation. angina, PAD
Peripheral Arterial Disease Study (ARTPER)[2]	October 2006-ongoing (at time of publication)	3786	Spain	Males and females, aged > 49 years, registered at 28 Primary Health Care centres in Barcelona.	Incidence of cardiovascular events and death	Two pairs of sequential BP readings recorded with sphygmomanometer (Welch Allyn, model Ds-66); results rounded to nearest 2 mmHg) with patient supine	Mean follow-up was 4 years	N/S	N/S	*Cardiovascular death:* Not defined.<br><br>*Non-fatal events*: Coronary artery disease: acute MI or angina, stroke or TIA, symptomatic aneurysm of abdominal aorta, vascular surgery, or cardiovascular morbidity
Chicago Walking and Leg Circulation Study (WALCS)[3]	1998-2000	740	USA	Patients without lower extremity peripheral artery disease who were recruited for the non-PAD comparator group.	Subclavian stenosis as a marker for total and cardiovascular disease mortality	Two pairs of sequential BP readings recorded using a 12-cm pneumatic cuff and a handheld Doppler probe (Nicolet Vascular Pocket Dop II, Golden, Colo) with patient supine	Mean follow-up was 4.8 years.	Patient history or use of BP lowering therapy	Patient history or use of oral antidiabetic drugs and/or insulin	*Cardiovascular death*: Any fatal cardiovascular cause.<br><br>*Non-fatal events*: MI, stroke, TIA, coronary or peripheral revascularisation, congestive heart failure, PAD, angina
Diabetes Alliance for Research in England (DARE)[4]	October 30th 2007-February 12th 2010	Type 1 or 2 diabetes: 727; Non-diabetic controls: 285	England	Males and females, with type 1 or 2 diabetes and non-diabetic controls, living in Devon	Inter-arm difference in BP and association with target organ disease and mortality	Four pairs of simultaneous BP readings recorded using a pair of automated sphygmomanometers (Omron 705IT; Omron Matsusaka, Japan) swapped after two readings, with patient seated	5 years. Median follow-up: 52 months	Use of BP lowering medication or recruitment SBP ≥ 140 mmHg or DBP ≥80 mmHg	Diagnosis of diabetes recorded in primary or secondary care diabetes registers	*Cardiovascular deaths*: MI, cardiac failure or ischemic stroke.<br><br>*Non-fatal events:* PAD, cardiovascular and cerebrovascular events, amputation

Study name	Period of patient recruitment /Duration of trial	Sample size	Country of origin	Eligibility criteria	Primary outcome measure	Blood pressure measurement method for IAD	Intended maximum duration of follow up	Definition of hypertension	Definition of diabetes	Definition of cardiovascular death and non-fatal cardiovascular event
Chronic Disease Detection and Management in the Elderly (Elderly Chinese)[5]	2006-2008	3133	China	Males and females, aged ≥ 60 years, living in a newly urbanized suburban town 30km from Shanghai	Predictive value of BP for cardiovascular morbidity and mortality	Two pairs (first pair discarded) of simultaneous BP readings recorded using a Vascular Profiler-1000 device (Omron, Kyoto, Japan) with patient supine	Followed up for vital status and cause of death until June 2011. Median follow-up: 4 years	SBP ≥140 mmHg, DBP ≥90 mmHg (average of 3 readings) or use of BP lowering drugs	Plasma glucose ≥7.0 mmol/L fasting or 11.1 mmol/L non-fasting, or use of antidiabetic agents	*Cardiovascular deaths:* Stroke, MI or other cardiovascular diseases, cerebrovascular disease and PAD
Epidemiology of dementia in Central Africa (EPIDEMCA)[6]	November 2011-December 2012	2002	Central African Republic/ Republic of Congo	Males and females, aged ≥ 65 years living in areas of Central African Republic and Republic of Congo	Diagnosis of dementia and Alzheimer's disease and associated risk factors	Two pairs of BP measurements recorded in each arm using standard mercury sphygmomanometer, as part of ABI protocol, with patients supine. BP rounded to nearest 5 mmHg	2-3 years	Self-reported BP lowering treatment; SBP ≥140 mmHg or DBP ≥90 mmHg	Self-reported or blood glucose >126 mg/dL fasting or >200 mg/dL in non-fasting	*Cardiovascular death:* Stroke, MI or other cardiovascular or cerebrovascular diseases – based on interview of relatives during verbal autopsy at follow-up. Non-fatal events: Stroke, MI, other heart disease
Fuencarral Health Center[7]	2003-2004	1361	Spain	Males and females, aged 60-79 years, with no known PAD	Low ABI and incidence of death due to cardiovascular causes	BP measured Doppler 8-MHz probe (Hadeco, Kawasaki, Japan) and calibrated mercury sphygmomanometer as part of ABI protocol with patient supine	Mean follow-up 49.8 months	SBP ≥140 mmHg, DBP ≥90 mmHg or use of BP lowering treatment	Baseline glucose ≥126 mg/dl (>7 mmol/L) on 2 occasions or use of antidiabetic agents	*Cardiovascular death:* Fatal stroke, MI, sudden death without other cause, death after vascular surgery or procedure, death attributed to heart failure, bowel or limb infarction, any other death not categorically attributed to a non-vascular cause *Non-fatal events*: MI, stroke or cardiovascular event
Heinz Nixdorf Recall Study[8]	2000-2003	4735	Germany	Males and females, aged 45-74 years, in an unselected urban population from the Ruhr area	Coronary artery calcium as predictor for fatal and non-fatal MI. Secondary endpoints included ABI as a stroke predictor factors	BP measured sequentially using Doppler probe (Logidop, Kranzbuhler, Germany) with patients supine	Mean follow up: 109 months	SBP >140mmHg or DBP >90mmHg	Existing diagnosis or use of anti-diabetic medication	*Cardiovascular death or non-fatal event:* First occurrence of MI based on symptoms, ECG signs, and enzymes, supported by necropsy if fatal

Study name	Period of patient recruitment /Duration of trial	Sample size	Country of origin	Eligibility criteria	Primary outcome measure	Blood pressure measurement method for IAD	Intended maximum duration of follow up	Definition of hypertension	Definition of diabetes	Definition of cardiovascular death and non-fatal cardiovascular event
Invecchiare in Chianti (InCHIANTI)[9]	August 1998-March 2000	1270	Italy	Males and females, aged ≥ 65 years, living in Greve and Bagno	Physiological factors influencing walking ability	Single pair of sequential BP readings using standard mercury sphygmomanometer, with patients supine. BP rounded to nearest 5 mmHg	N/S	Self-reported, existing, recorded diagnosis or use of BP lowering medication or SBP ≥140 mmHg or DBP ≥90 mmHg	Self-reported, existing recorded diagnosis, or use of anti-diabetic medication, or fasting glucose >7.0 mmol/L	*Cardiovascular death:* Not defined. *Non-fatal events:* Diagnosis of heart disease, MI or angina, stroke or TIA
Kinmen Health Survey[10]	2002-2012	1329	Kinmen (Republic of China)	Community living individuals, aged ≥ 40 years	Association between ABI and brachial-ankle pulse and mortality	Simultaneous BP readings were recorded. Device or measurement position not stated	Median follow up: 10 years	N/S	N/S	*Cardiovascular death*: "Death by cardiovascular cause" *Non-fatal events:* Not described
Lifestyle Interventions and Independence for Elders (LIFE) study[11]	2010-2011/ 2.6 years	1635	USA	Ambulant community dwelling individuals, aged 70-89 years with a sedentary lifestyle (<20min per week physical activity)	Major mobility disability<br><br>Secondary: Association between ABI and cognitive function	Two pairs of sequential BP measurements recorded in each arm using handheld Doppler, with patients supine	2 years	Self-reported or measurement	Self-reported	*Cardiovascular fatal or non-fatal events:* MI, angina, stroke or TIA, carotid artery disease, congestive heart failure or PAD requiring hospitalisation, outpatient revascularisation for PAD, ruptured abdominal aortic aneurysm
Limburg PAOD Study[12]	N/S	3649	The Netherlands	Males and females, from 18 General Practice clinics, aged 40-75 years, in Limburg	Progressive limb ischaemia, non-fatal cardiovascular morbidity and mortality	BP measured in both arms using pocket Doppler device (Huntleigh Mini Dopplex D500, 8Mhxz) and a sphygmomanometer	Mean follow up: 7.2 years	Existing recorded diagnosis	Existing recorded diagnosis	*Cardiovascular deaths:* MI, sudden deaths, strokes, aortic aneurysms and death due to PAD complications *Non-fatal events:* MI, angina, stroke, aortic aneurysm., PAD, vascular surgery or intervention, amputations due to PAD
Improving interMediAte RisK management (MARK) study[13]	N/S	2688	Spain	Males and females living in 3 regions of Spain, aged 35-74 years. Free of atherosclerotic disease, with an intermediate cardiovascular risk (10-year coronary risk of 5-15% or vascular death risk of 3-5%) selected at random	Incidence of vascular events	Three pairs of BP measurements in each arm, using an OMRON 705, with patients seated	10 years	Patient reported, or use of BP lowering medications or SBP ≥140mmHg or DBP ≥90mmHg	Patient reported, or use of antidiabetic treatment or fasting glucose ≥ 126 mg/dL	*Cardiovascular death:* not defined<br><br>*Non-fatal events*: Stroke or TIA, MI, angina, or revascularisation procedure

Study name	Period of patient recruitment /Duration of trial	Sample size	Country of origin	Eligibility criteria	Primary outcome measure	Blood pressure measurement method for IAD	Intended maximum duration of follow up	Definition of hypertension	Definition of diabetes	Definition of cardiovascular death and non-fatal cardiovascular event
Action for Health in Diabetes (Look AHEAD)[14]	June 2001-March 2004	479	USA	Overweight and obese individuals with type 2 diabetes aged 45-76 years, and had a body mass index, 25 kg/m2, or ≥27 kg/m2 if taking insulin	A composite cardiovascular outcome: cardiovascular death, non-fatal MI, non-fatal stroke, hospitalized angina<br><br>Secondary: Cognitive function	Two pairs of sequential BP measurements recorded in each arm, using continuous wave Doppler with a standard mercury sphygmomanometer, with patients supine	4-5 year follow up	SBP ≥140 mmHg, ≥DBP > 90 mmHg or taking BP lowering medication	Self-reported verified from medical records, current treatment, or fasting glucose of ≥126 mg/dL	*Cardiovascular death:*<br>MI, congestive heart failure, death after cardiovascular intervention, surgery or due to arrhythmia, stroke, presumed cardiovascular death, rapid unexplained cardiovascular death.<br>*Non-fatal events:*<br>Stroke, MI, angina, coronary artery bypass grafting or percutaneous coronary intervention, congestive heart failure, carotid endarterectomy, peripheral arterial bypass or angioplasty
Men born in 1914.[15]	1982-1983	474	Sweden	Males, born in even months in 1914 and residing in Malmö in 1982–1983	Prevalence of PAD and cardiovascular mortality	Two BP measurements, recorded using mercury-in-silastic strain gauges, with patients supine	14.3 years. Median: 13.2 years	SBP ≥160mmHg, DBP ≥100mmHg or use of BP lowering medications	History of diabetes or fasting blood glucose ≥ 6.1 mmol/L	*Cardiovascular death:*<br>MI or death from ischemic heart disease<br>*Non-fatal events:*<br>MI
Multi Ethnic Study of Atherosclerosis (MESA)[16]	2000-2002	6743	USA	Males and females, aged 45-84 years, free of clinical cardiovascular diagnoses at baseline	Association of subclavian stenosis with markers of cardiovascular disease	Single pair of sequential BP measurements, using hand-held Doppler instrument and 5-mHz probe, with patients supine	N/S	Self-reported history with use of BP lowering medications,<br>or SBP ≥140mmHg or DBP ≥ 90mmHg	Fasting blood glucose ≥126 mg/dl or use of oral hypoglycemic agents or insulin	*Cardiovascular death*:<br>Death due to atherosclerotic coronary heart disease, stroke, other cardiovascular disease.<br>*Non-fatal* events:<br>Stroke, TIA, MI, angina, revascularisation procedure
Mid Devon Hypertension cohort[17]	9 November 1999 to 17 June 2002	230	England	Males and females, receiving treatment for hypertension in rural general practices in Devon	Cardiovascular events and deaths from all causes	Single pair of sequential BP measurements, using standard calibrated mercury sphygmomanometer, with patients seated	11.4 years. Median follow up: 9.8 years	SBP ≥160 mmHg or DBP ≥100 mmHg or ≥140/90 mmHg with target organ damage, diabetes or coronary heart disease risk score > 15%	N/S	*Cardiovascular death*.<br>Not defined<br>*Non-fatal events*:<br>Stroke, TIA, MI or angina
Mid Devon Pilot study[18]	May 1994-Ocober 1995	280	England	Males and females, aged 19-88 years (median age: 60 years), attending a general practice for re-registration medicals	Inter-arm difference, new diagnosis of angina, MI, cerebrovascular event or death	Single pair of sequential BP measurements, using standard calibrated mercury sphygmomanometer, with patients seated	5.6 years	N/S	N/S	*Cardiovascular fatal and non-fatal events:*<br>Cardiovascular disease-related causes. Ischaemic heart disease events and cerebrovascular events

Study name	Period of patient recruitment /Duration of trial	Sample size	Country of origin	Eligibility criteria	Primary outcome measure	Blood pressure measurement method for IAD	Intended maximum duration of follow up	Definition of hypertension	Definition of diabetes	Definition of cardiovascular death and non-fatal cardiovascular event
San Diego Population Study[19]	1994-1998	2404	USA	Males and females, aged 29-91 years, attending a clinic for assessment of PAD and venous disease	Prevalence of PAD	Two pairs of BP measurements, using a continuous-wave Doppler ultrasound, with patients supine	N/S	SBP ≥140 mmHg or DBP ≥ 90 mmHg or use of BP lowering medications	Self-reported or use of antidiabetic medications	*Cardiovascular death:* not defined *Non-fatal events:* MI, stroke, angina, coronary angioplasty or bypass graft, or carotid endarterectomy
Second Manifestations of ARTerial disease (SMART) study[20]	January 2002 – February 2014	7344	The Netherlands	Males and females, aged 18-80 years, referred to University Medical Center Utrecht, for treatment of clinically manifest vascular disease or cardiovascular risk factors	3-point MACE (combination of non-fatal myocardial infarction, non-fatal stroke and death from vascular disease), total mortality and vascular mortality	Single pair of sequential BP measurements, using a Vasoguard Doppler probe, with patients supine	Mean follow-up:5.9 years	Blood pressure >140/90 mmHg at baseline or the use of blood pressure lowering medication.	Recorded diagnosis, self-reported diagnosis, use of blood glucose lowering medication, or fasting glucose >7 mmol/L at recruitment combined with initiation of glucose lowering medication within first year of follow-up. Type 1 diabetes excluded.	*Cardiovascular death:* Death from stroke, MI, congestive heart failure, rupture of abdominal aortic aneurysm or vascular death from other causes *Non-fatal events:* Stroke (infarction or haemorrhagic), MI, retinal infarction, heart failure (see published data supplement for full definitions)[20]
Surrogate markers for Micro- and Macrovascular hard endpoints as Innovative diabetes tools (SUMMIT)[21]	November 2010 – June 2013	596	England	Adults over 18 with and without diabetes and/or cardiovascular disease		6 pairs of simultaneous BP readings using two Omron 705 devices swapped after 3 readings, with patients supine	N/S	Self-reported history of hypertension	HbA1c ≥ 48 mmol/mol	*Cardiovascular death:* Fatal MI
Viborg Women Cohort (ViWoCo)[22]	October 2011- January 2013	1474	Denmark	Females born in 1936, 1941, 1946 and 1951 living in the Municipal of Viborg, Denmark	Presence of cardiovascular disease and diabetes mellitus	One pair of simultaneous BP readings, using Omron M2 devices, with patients supine, rounded to nearest 2mmHg	Median follow-up 3.3 years	SBP ≥160 mmHg or DBP ≥100 mmHg	HbA1c ≥ 48 mmol/mol	*Cardiovascular death:* Fatal event as below *Non-fatal event:* MI or ischaemic stroke leading to hospitalisation
Vietnam Experience Study[23]	1986	4419	USA	Male US army veterans who participated in the Vietnam war	Inter-arm differences, all-cause and cardiovascular mortality	Two pairs of sequential BP measurements, using standard mercury sphygmomanometer, with patients seated	15 years	SBP ≥140 mmHg, DBP ≥90 mmHg or use of BP lowering medication	Fasting plasma glucose ≥ 7.0 mmol/l and/or use of medication for diabetes	*Cardiovascular death:* Death due to major cardiovascular disease.

ABI = ankle-brachial index, BP = BP, DBP = diastolic BP, IHD = ischaemic heart disease, MI = myocardial infarction, N/S = not stated, PAD = peripheral arterial disease, SBP = systolic BP
TIA = transient ischaemic attack, ECG = electrocardiogram

References to included studies

1. Clark CE, Taylor RS, Butcher I, et al. Inter-arm blood pressure difference and mortality: a cohort study in an asymptomatic primary care population at elevated cardiovascular risk. *British Journal of General Practice* 2016;66(5):241-2. doi: 10.3399/bjgp16X684949
2. Alzamora MT, Baena-Díez JM, Sorribes M, et al. Peripheral Arterial Disease Study (PERART): Prevalence and predictive values of asymptomatic peripheral arterial occlusive disease related to cardiovascular morbidity and mortality. *BMC public health* 2007;7(1):1-7. doi: 10.1186/1471-2458-7-348
3. McGrae McDermott M, Greenland P, Liu K, et al. Leg symptoms in peripheral arterial disease: Associated clinical characteristics and functional impairment. *JAMA* 2001;286(13):1599-606. doi: 10.1001/jama.286.13.1599
4. Clark CE, Steele AM, Taylor RS, et al. Inter-arm blood pressure difference in people with diabetes: measurement and vascular and mortality implications: a cohort study. *Diabetes Care* 2014;37:1-8.
5. Sheng CS, Liu M, Zeng WF, et al. Four-Limb Blood Pressure as Predictors of Mortality in Elderly Chinese. *Hypertension* 2013;61(6):1155-60.
6. Guerchet M, Mbelesso P, Ndamba-Bandzouzi B, et al. Epidemiology of dementia in Central Africa (EPIDEMCA): protocol for a multicentre population-based study in rural and urban areas of the Central African Republic and the Republic of Congo. *SpringerPlus* 2014;3:338. doi: 10.1186/2193-1801-3-338 [published Online First: 2014/07/22]
7. Lahoz C, Barrionuevo M, Garcia-Fernandez T, et al. Cardiovascular morbidity-mortality associated to ankle-brachial index in the general population. [Spanish]. *Revista Clinica Espanola* 2014;214(1):1-7.
8. Erbel R, Mohlenkamp S, Moebus S, et al. Coronary risk stratification, discrimination, and reclassification improvement based on quantification of subclinical coronary atherosclerosis: the Heinz Nixdorf Recall study. *J Am Coll Cardiol* 2010;56(17):1397-406. doi: 10.1016/j.jacc.2010.06.030 [published Online First: 2010/10/16]
9. Clark CE, Thomas D, Llewellyn DJ, Ferrucci L, Bandinelli S, Campbell JL. Systolic inter-arm blood pressure difference and risk of cognitive decline in older people: a cohort study. *British Journal of General Practice*. 2020:bjgp20X709589
10. Chuang SY, Sung SH, Cheng HM, et al. Ankle-brachial index and brachial-ankle pulse wave velocity jointed to predict mortality in a community study. *European Heart Journal* 2015;36:1015-16.
11. Espeland MA, Newman AB, Sink K, et al. Associations Between Ankle-Brachial Index and Cognitive Function: Results From the Lifestyle Interventions and Independence for Elders Trial. *Journal of the American Medical Directors Association* 2015;16(8):682-89.
12. Hooi JD, Stoffers HE, Kester AD, et al. Risk factors and cardiovascular diseases associated with asymptomatic peripheral arterial occlusive disease: The Limburg PAOD Study. *Scand J Prim Health Care* 1998;16:177-82. doi: 10.1080/028134398750003142
13. Marti R, Garcia-Regalado N, Garcia-Gil M, et al. Improving interMediAte risk management. MARK study. *BMC Cardiovascular Disorders* 2011;11:61.
14. Espeland MA, Beavers KM, Gibbs BB, et al. Ankle-brachial index and inter-artery blood pressure differences as predictors of cognitive function in overweight and obese older adults with diabetes: Results from the Action for Health in Diabetes movement and memory study. *International Journal of Geriatric Psychiatry* 2015;30(10):999-1007.
15. Ogren M, Hedblad B, Engstrom G, et al. Prevalence and prognostic significance of asymptomatic peripheral arterial disease in 68-year-old men with diabetes. Results from the population study 'Men born in 1914' from Malmo, Sweden. *European Journal of Vascular & Endovascular Surgery* 2005;29(2):182-9.
16. Bild DE, Bluemke DA, Burke GL, et al. Multi-Ethnic Study of Atherosclerosis: Objectives and Design. *American Journal of Epidemiology* 2002;156(9):871-81.

17. Clark CE, Taylor RS, Shore AC, et al. The difference in blood pressure readings between arms and survival: primary care cohort study. *BMJ* 2012;344:e1327.
18. Clark CE, Powell RJ. The differential blood pressure sign in general practice: prevalence and prognostic value. *Family Practice* 2002;19(5):439-41.
19. Wassel CL, Loomba R, Ix JH, et al. Family History of Peripheral Artery Disease is associated with Prevalence and Severity of Peripheral Artery Disease: The San Diego Population Study (SDPS). *Journal of the American College of Cardiology* 2011;58(13):1386-92. doi: 10.1016/j.jacc.2011.06.023
20. Kranenburg G, Spiering W, de Jong PA, et al. Inter-arm systolic blood pressure differences, relations with future vascular events and mortality in patients with and without manifest vascular disease. *International journal of cardiology* 2017;244:271-76. doi: 10.1016/j.ijcard.2017.06.044
21. Clark CE, Casanova F, Gooding K, et al. Inter-arm blood pressure difference and arterial stiffness. *Journal of Hypertension* 2014;32(eSuppl A):e30.
22. Dahl M, Frost L, Søgaard R, et al. A population-based screening study for cardiovascular diseases and diabetes in Danish postmenopausal women: acceptability and prevalence. *BMC cardiovascular disorders* 2018;18(1):20. doi: 10.1186/s12872-018-0758-8
23. White J, Mortensen LH, Kivimaki M, et al. Interarm differences in systolic blood pressure and mortality among US army veterans: aetiological associations and risk prediction in the Vietnam experience study. *EurJPrevCardiol* 2014;21(11):1394-400.

Results for SCORE analyses

All participants eligible for the SCORE analyses came from countries covered by the low-risk (as opposed to the high risk) risk algorithms, which were, consequently, used throughout. Mean European SCORE values for 18,017 eligible participants free of pre-existing cardiovascular disease were significantly higher when calculated using the higher arm blood pressure in comparison to the lower arm reading (2.6 (SD 2.8) vs. 2.3 (2.4); P <0.001). However, AIC values for cardiovascular death modelling with SCORE showed no difference between arms (higher arm AIC 2419, lower arm 2421; difference 2; P =0.269). Harrell's C-statistics suggested better modelling of events with the higher arm (higher arm 0.749, lower arm 0.743), but the difference was not significant on likelihood ratio testing (P = 0.205).

Reclassification from below to above guideline-recommended risk thresholds, when higher rather than lower arm blood pressures were used, occurred for 7.7% of participants with SCORE (P <0.001; Table S2); results were similar where mean of more than one pair of blood pressure readings was available.

For participants with a systolic IAD ≥ 10 mmHg, based on a single pair of readings, reclassification, from below to above guideline-recommended risk thresholds within this subgroup, was observed for 16.3% with SCORE (P <0.001l; Table S2).

Table S2 - Reclassification across guideline recommended risk thresholds for SCORE cardiovascular mortality score

Threshold or category[#]	N	N (%) above threshold lower arm BP	N (%) above threshold higher arm BP	N (%) reclassified	p-value
**PRIMARY ANALYSES BASED ON FIRST PAIR OF BP READINGS**					
Low risk: <1%	5,350			N/A	
Moderate risk: 1% to <5%	10,282	9,636 (53.5%)	10,282 (57.1%)	646 (3.6%)	
High risk: 5% to <10%	1,901	1,344 (7.5%)	1,901 (10.6%)	557 (3.1%)	
Very high risk: ≥10%	484	295 (1.6%)	484 (2.7%)	189 (1.1%)	
Total across categories	18,017	11,275 (62.6%)	12,667 (70.3%)	1,392 (7.7%)	<0.001
**SENSITIVITY ANALYSES BASED ON MEAN BP READINGS FOR PARTICIPANTS WITH MORE THAN ONE PAIR OF READINGS**					
Low risk: <1%	1,346			N/A	
Moderate risk: 1% to <5%	2,771	2,559 (55.6%)	2,771 (59.2%)	172 (3.7%)	
High risk: 5% to <10%	489	346 (7.4%)	489 (10.5%)	143 (3.1%)	
Very high risk: ≥10%	73	45 (1%)	28 (0.6%)	73 (1.6%)	
Total across categories	4,679	2,950 (59.2%)	3,288 (70.3%)	388 (8.3%)	<0.001
**SENSITIVITY ANALYSES FOR PARTICIPANTS WITH SYSTOLIC INTER-ARM BP DIFFERENCE ≥ 10 mmHg**					
Low risk: <1%	1,191			N/A	
Moderate risk: 1% to <5%	3,058	2,684 (52.2%)	3,058 (59.4%)	374 (7.3%)	
High risk: 5% to <10%	708	357 (6.9%)	708 (13.8%)	351 (6.8%)	
Very high risk: ≥10%	188	74 (1.4%)	188 (3.7%)	114 (2.2%)	
Total across categories	5,145	3,115 (60.5%)	3,954 (76.9%)	839 (16.3%)	<0.001

BP = systolic blood pressure; # 10 year risk score percentages
p-values for chi-square tests of classification according to lower and higher reading arms

Data sources and funding acknowledgements for data contributing to the INTERPRESS-IPD Collaboration

The following collaborating authors contributed data to the INTERPRESS-IPD Collaboration and contributed to the final manuscript: Christopher E Clark *(Mid Devon cohorts)*; Ji-Guang Wang: *Elderly Chinese*; *Vietnam Experience Study*: James White; *DARE:* Andrew Hattersley; *AAA:* Jackie Price; *INCHIANTI:* Luigi Ferrucci; *Heinz Nixdorf Recall Study*: Raimund Erbel; *SMART*: Jan Westerink; *San Diego Population Study*: Michael H Criqui; *Fuencarral Health Center*: Carlos Lahoz; *ARTPER*: Maria Teresa Alzamora; *EPIDEMCA*: Maëlenn Guerchet; *MESA*: Robyn L McClelland; *LIFE & WALCS*: Mary McDermott; *Limburg PAOD Study*: Henri Stoffers; *Men born in 1914:* Gunnar Engström; *Look AHEAD*: Mark Espeland; *Kinmen Health Survey*: Shao-Yuan Chuang; *ViWoCo:* Marie Dahl; *SUMMIT*: Angela C Shore; *MARK Study*: Rafel Ramos Blanes

Funding for the cohorts contributing to the INTERPRESS-IPD Collaboration is also acknowledged as follows:

Mid Devon cohorts: Supported by the Scientific Foundation Board of the Royal College of General Practitioners (grant No SFB-2009-06), the South West GP Trust, and the NIHR Peninsula Collaboration for Leadership in Applied Health Research and Care (PenCLAHRC)

Elderly Chinese: Supported by the National Natural Science Foundation of China (30871360, 30871081, 81170245, and 81270373), the Ministry of Science and Technology (a grant for China-European Union collaborations [1012]) and the Ministry of Education (NCET-09-0544), Beijing China, the Shanghai Commissions of Science and Technology (11QH1402000) and Education (the Dawn project 08SG20), the Shanghai Bureau of Health (2009Y111 and XBR2011004), and Shanghai Jiaotong University School of Medicine

Vietnam Experience Study: Mortality surveillance of the cohort in the post-service VES was funded by the National Center for Environmental Health in Atlanta, USA. Dr White is funded by the Centre for the Development and Evaluation of Complex Interventions for Public Health Improvement, a UKCRC Public Health Research: Centre of Excellence. Funding from the British Heart Foundation, Cancer Research UK, Economic and Social Research Council (ESRC RES-590-28-0005), Medical Research Council, the Welsh Assembly Government and the Wellcome Trust (WT087640MA), under the auspices of the UK Clinical Research Collaboration, is gratefully acknowledged.

DARE: Supported by the NIHR Exeter Clinical Research Facility, the Diabetes Research Network and the Wellcome Trust

AAA: Supported by the British Heart Foundation and the Chief Scientist's Office of the Scottish Government

INCHIANTI: NIHR (CEC: Clinical Lectureship award). the James Tudor Foundation, the Mary Kinross Charitable Trust, the Halpin Trust, PenCLAHRC), the National Institute on Aging/National Institutes of Health (NIA/ NIH) (award number RF1AG055654), the Alan Turing Institute under the Engineering and Physical Sciences Research Council grant EP/N510129/1. the Intramural Research Program of the NIA/NIH, Baltimore MD, US.

Heinz Nixdorf Recall Study: Supported by the Heinz Nixdorf Foundation, Germany.

SMART: Supported by the University Medical Center Utrecht

San Diego Population Study: Supported by the National Heart, Lung, and Blood Institute, NIH (grant 53487), and the NIH General Clinical Research Center Program (grant M01 RR0827), Bethesda, Maryland

ARTPER: Supported by the program of Promotion in the Biomedical Investigation and Health Sciences from the Carlos III Health Institute of the Spanish Health and Consumption Ministry [PI070403]

EPIDEMCA: Supported by the French National Agency (ANR), (ANR-09-MNPS-009-01 grant) and AXA Research Fund (grant 2012 – Project – Public Health Institute (Inserm) – PREUX Pierre-Marie).

MESA: Supported by contracts 75N92020D00001, HHSN268201500003I, N01-HC-95159, 75N92020D00005, N01-HC-95160, 75N92020D00002, N01-HC-95161, 75N92020D00003, N01-HC-95162, 75N92020D00006, N01-HC-95163, 75N92020D00004, N01-HC-95164, 75N92020D00007, N01-HC-95165, N01-HC-95166, N01-HC-95167, N01-HC-95168 and N01-HC-95169 from the National Heart, Lung, and Blood Institute, and by grants UL1-TR-000040, UL1-TR-001079, and UL1-TR-001420 from the National Center for Advancing Translational Sciences (NCATS).

LIFE: Supported by a National Institutes of Health (NIH)/ National Institute on Aging Cooperative Agreement (number UO1 AG22376) and a supplement from the National Heart, Lung and Blood Institute (number 3U01AG022376-05A2S). The research is also partially supported by the Claude D Pepper Older Americans Independence Centers at the University of Florida (grant number 1 P30 AG028740), Wake Forest University (grant number 1 P30 AG21332), Tufts University (grant number 1 P30 AG031679), University of Pittsburgh (grant number P30 AG024827), and Yale University (grant number P30 AG021342), and the NIH/National Center for Research Resources (NCRR) Clinical and Translational Science Award (CTSA) at Stanford University (number UL1 RR025744). The Tufts University LIFE field center is also supported by the Boston Rehabilitation Outcomes Center (grant number 1R24HD065688-01A1).

WALCS: Supported by grant R01-58099 from the National Heart, Lung, and Blood Institute and grant RR-00048 from the National Center for Research Resources.

Limburg PAOD Study: Supported by a research grant of the Netherlands Organization for Scientific Research (900-715.154), the Dutch ‘‘Praeventiefonds’’ (28-1323) and a research grant from the Netherlands Heart Foundation (92.170).

Men born in 1914: Supported by the Swedish Heart-Lung foundation and Swedish Research Council

Look AHEAD: Supported by the National Institute on Aging, National Institutes of Health, Department of Health and Human Services, R-01AG033087-01 and R-01AG033087-04S1. The Action for Health in Diabetes is supported through the following cooperative agreements from the National Institutes of Health: DK57136, DK57149, DK56990, DK57177, DK57171, DK57151, DK57182, DK57131, DK57002, DK57078, DK57154, DK57178, DK57219, DK57008, DK57135, and DK56992.

ViWoCo: Supported by the Health Research Foundation of Central Denmark Region; Institute of Clinical Medicine, Aarhus University, Denmark; Regional Hospital Central Jutland Research Foundation, The Foundation of Rosa and Asta Jensen, Viborg Regional Hospital, Denmark and Odd Fellow, Viborg, Denmark.

SUMMIT: Supported by the Danish Research Foundation, The Danish innovation foundation and IMI (grant no: 115006)

MARK Study: Supported by grants funded by: the Spanish Ministry of Science and Innovation (MICINN) and Carlos III Health Institute/European Regional Development Fund (ERDF) (MICINN, ISCIII/FEDER) (Red RedIAPP RD06/0018, Research Groups: RD06/0018/2009, RD06/0018/0045/RD06/0018/0027) and by the Fondo de Investigación Sanitaria (FIS PI10/01088, PI10/02077, PI10/02043) and Regional Health Management of the Castilla y León (GRS 635/A/11).
